# Keel bone fractures in Danish laying hens: Prevalence and risk factors

**DOI:** 10.1371/journal.pone.0256105

**Published:** 2021-08-13

**Authors:** Ida C. N. Thøfner, Jan Dahl, Jens Peter Christensen

**Affiliations:** 1 Department of Veterinary & Animal Sciences, University of Copenhagen, Copenhagen, Denmark; 2 Danish Agriculture & Food Council, Copenhagen, Denmark; University of Life Sciences in Lublin, POLAND

## Abstract

Keel bone fractures (KBF) in commercial poultry production systems are a major welfare problem with possible economic consequences for the poultry industry. Recent investigations suggest that the overall situation may be worsening. Depending on the housing system, fracture prevalences exceeding 80% have been reported from different countries. No specific causes have yet been identified and this has consequently hampered risk factor identification. The objective of the current study was to investigate the prevalence of KBF in Danish layer hens and to identify risk factors in relation to KBF in all major productions systems, including parent stock production. For risk factor identification, production data from the included flocks was used. In total, 4794 birds from 40 flocks were investigated at end-of-lay. All birds were euthanized on farm and underwent inspection and palpation followed by necropsy. All observations were recorded and subsequently analysed using the SAS statistical software package. In flocks from non-caged systems, fracture prevalence in the range 53%-100%, was observed whereas the prevalence in flocks from enriched cages ranged between 50–98%. Furthermore, often multiple fractures (≥4) were observed in individual birds (range 5–81% of the birds with fractures) depending on the flock. The localization of the fractures at the distal end of the keel bone is highly consistent in all flocks (>96%). Macroscopically the fractures varied morphologically from an appearance with an almost total absence of callus, most frequently observed in caged birds, to large callus formations in and around the fracture lines, which was a typical finding in non-caged birds. Despite being housed under cage-free conditions, parent birds had significantly fewer fractures (all flocks were 60 weeks old) per bird, than other birds from cage-free systems. The body weight at end-of-lay had an effect on the risk of having fractures, heavy hens have significantly fewer fractures at end-of-lay. The older the hens were at onset of lay, the lower was the flock prevalence at end-of-lay. Additionally, the daily egg size at onset of lay was of importance for the risk of developing fractures, the production of heavier eggs initially, resulted in higher fracture prevalence at depopulation. The odds ratio of body weight, (+100 g) was 0.97, age at onset of lay (+1 week) was 0.87 and daily egg weight at onset (+1 gram) was 1.03. In conclusion, the study demonstrated a very high prevalence of KBF in hens from all production systems and identified hen size, age at onset of lay and daily egg weight at onset of lay to be major risk factors for development of KBF in the modern laying hen. Further research regarding this is warranted to strengthen the longevity and enhance the welfare of laying hens.

## Introduction

Currently, keel bone fractures (KBF) are considered the most important welfare problem for layer hens [1,2]. Within the last 20 years, KBF in egg laying hens have significantly increased in prevalence, globally. The reported prevalences are alarmingly high in both caged and non-caged production systems, with up to 97% in non-caged systems [3–11].

Despite considerable efforts, so far no one has been able to propose a plausible cause of these fractures. The current opinion is that the fractures are caused by trauma related to impact of the bird with the furniture and equipment in the hen house [4,12–18]. This is, however, difficult to understand when the prevalences in caged birds are at comparable levels to alternative systems where hens can move freely [4,7]. Based on observed features of the fractures, detected using CT scanning and different histopathological techniques of fractured keels, there are strong indications that the fractures are not due to external trauma [19]. In this study, inflammatory cell types normally accumulating in response to trauma, either in the bone or in the soft tissues around the fractures, could not be identified. It was also observed that the fractures seem to develop from the inside resembling greenstick fractures in young individuals and/or stress fractures. No apparent healing of the fractures, involving e.g. osteoblasts along the fracture lines was observed.

Identification of risk factors is an important step in the understanding of the true cause of fracture development. However, the attemps to identify the exact cause and predisposing risk factors for the fractures to develop may have been hampered by the lack of a clear distinction between keel bone fractures and keel bone deviations, with overlapping nomenclature (e.g. keel bone deformities, keel bone damage, keel bone disorders, crooked keels etc.). Sometimes KBF and deviations were even recorded seperately but pooled in the data analysis as keel bone damage [20]. The consequenses of not having a clear definition and nomenclature will undoubtedly contribute to the difficulties in identifying the pathogenesis since deviations and fractures may have different causes, thus diffferent pathogeneses. Several published studies indicate that the S-shaped deviation of the ventral rim (of the middle third) of the keel is very likely to be associated with perches and perching behaviour (e.g. resting the keel on the perch, perch design etc.) [14,21,22].

Another factor that may have influenced the identification and observed levels of KBF is the diagnostic methods used. Diagnosis of keel bone abnormalities have, to a great extent, relied on palpation [3,23–26] whereas others have used diagnostic imaging like radiography, CT-scanning, and ultrasonography for detection of KBF and deviations in the birds [10,16,27–32]. However, as shown previously, the method used may influence the outcome of prevalence estimation significantly [19].

Decreased bone health and layer fatigue/osteoporosis have been suggested as risk factors contributing to fracture development in keel bones [16,33–35]. In a recent study conducted in organic layer flocks (52–73 weeks old) in several European countries, the authors observed that housing system (aviary *vs*. floor systems), absence of natural daylight in the hen house, higher proportions of underweight hens and higher laying performance were significantly associated with keel bone damage [20]. In this study, keel bone observations were obtained by palpation and inspection of live birds and keel bone fracture observations were pooled together with keel bone deviations. Differences in furniture and layout of aviaries have also been suggested as contributing risk factors of keel bone disorders (i.e. fractures, deviations and soft tissue damage) [11], but direct association to the fractures alone remains unclear. Early onset of lay has been proposed as a risk factor for the development of keel bone fracture [36]. High laying performance has also been proposed as a risk factor [20,37], whereas others have found no or negative association between laying performance and KBF [11,34,36]. Regarding feed management in relation to fracture development, it has been proposed that the composition of fatty acids in the diet may contribute to keel bone health [38,39] whereas phosphorus supplementation was found to be of no significance [20]. Furthermore, some association with the genetic background of the hens has been suggested [11,30,37,40–42]. The coincidence of fracture development and late ossification in hens during peak production also renders the maturation of the keel bone as a potential risk factor for fracture development [19,43].

The majority of the fractures develop during weeks 25–54 of life [17,22,37,44,45], thus indicating a relationship between the egg laying process and ossification, changing from a cartilagenous keel, via immature bone to the mature keel bone. The ossification of the caudal part of the keel bone is not complete before the birds are 35–40 weeks old [46,47]. It has been proposed that after the onset of lay there is a significant load on the immature bones, including the keel, with regards to positioning of the egg, Ca^2+^-mobilization for the egg shell and ossification of the immature keel bone [43,48]. Thøfner et al. [19] observed a highly consistent and greenstick-like appearance of KBF, which suggests that the ossification of the keel bone may play a key role in KBF development, as greenstick fractures are characterized by occurrence in immature bones of young individuals [49].

The objectives of the current study were to determine the prevalences of KBF in Danish layer hens from different production systems at the end of lay and to identify possible risk factors for the occurrence of KBF in production settings.

## Materials and methods

### Study population

From 2016 to 2019, a total of 4756 females and 38 males collected from 40 flocks of layers were included in the study (Table 1), originating from the common production systems in Denmark, namely enriched cages (n = 11), or from non-caged systems: barn housed (n = 15) and organic/free range (n = 11). Three parent stock flocks were also included.

**Table 1:** Overview of the flocks and birds in the different production systems.

Production type (n = 40)	No. flocks	No. females	No. males
Enriched cages	11	1322	-
Barn housed/Aviary	15	1782	-
Organic/Free range	11	1316	-
Parent stock	3	336	38

The free-range flocks consisted primarily of organic flocks. The free-range flocks included were, due to housing restrictions imposed during an outbreak of avian influenza in the winter of 2016–2017, reclassified as free-range, despite originally (at time of replacement) being classified as organic.

From each flock, 120 hens were collected on the farm immediately after euthanasia at the planned depopulation time at the end of the laying period. In the parent stock flocks, male birds were collected in the approximate male-to-female ratio as housed. The euthanized hens were transported to the poultry section facilities at University of Copenhagen where they underwent full post mortem investigation.

Information on the hen line was available for 29 flocks (Table 2), 17 of these flocks had Lohmann LSL Classic birds. Due to the evident skewness in the hen line distribution, no further analysis was done in relation to the hen line.

**Table 2:** Distribution of the hen lines in 29 flocks out of the study population.

Hen line (n = 29)	No. flocks	KBF Prevalence (%)
**Bovans Brown**	1	88.24
**Dekalb White**	1	99.16
**Hisex**	2	98.75
**Lohmann Brown**	2	79.32
**Lohmann Brown-Lite**	3	93.84
**Lohmann LSL Classic**	17	86.55
**Lohmann LSL Parent Stock**	3	81.55

### Post mortem evaluation

The bodyweight of all hens was recorded. Prior to necropsy, inspections and palpation of the keel to identify deviations and fractures were performed. Palpation and subsequent scoring followed the protocols of Käppeli et al. [5] and Hinrichsen et al. [50] with the modification to include palpation of the visceral part of the caudal 3–5 cm of the keel bone. This was done by inserting the index finger under the rib curvature at the apex of the keel. Keel deviation was scored according to the above-mentioned protocols (score 1: Deviation x>1cm; score 2: Deviation 0.5<x≤1 cm; and score 3: Deviation x≤0.5 cm). After the palpation, the skin was removed from the pectoral muscles and keel bone deviation was re-assessed using the above-mentioned scorings systems. In addition, the localisation of the deviation was recorded as described by Thøfner et al. [19] (cranial, middle, and/or caudal section of the keel bone). Subsequent necropsy, included characterization of KBF by the following parameters: Presence/absence; number of fractures (0, 1, 2, 3, ≥4); localization of fracture [19]; new/old; callus formation (no, minimal, abundant, fresh fractures/non-union); localization of callus. Two experienced poultry pathologists performed all procedures in all birds.

### Production data retrieval

Production data from the majority of the flocks (n = 24) in this study was retrieved from a centrally administered database (E-kontrol) where production data from the majority of the Danish egg producers are recorded and stored. The data retrieved from the database were hen line, age, egg yield, and egg weight at six predefined time points during the laying cycle (Table 3). The time points were onset of lay, peak production, end-of-lay, and flock age- namely 45, 50 or 70 weeks old.

**Table 3:** Information obtained from production database records.

Time point in production cycle	Age in weeks	Laying performance in percent	Average egg weight in grams
Onset of lay	+	+	+
Peak production	+	+	+
End-of-lay	+	+	+
Week 45	n/a ^1^	+	+
Week 50	n/a	+	+
Week 70	n/a	+	+

^1^ Not applicable.

### Data management and statistical analysis

All observations were entered for each flock into a spreadsheet (Microsoft ® Excel ® 2016, Microsoft Cooperation) and one of the two pathologists proofed all sheets/flocks. After proofing, the data were ready for further analysis. If data cells were found to be empty, the observation/information for the specific variable was assigned as missing. Birds with missing values in analysis of specific variables were excluded from the specific analysis, resulting in n-values below the total number of investigated birds. The male birds were only included in the fracture identification analysis, where palpation and necropsy agreement were compared.

Agreement between palpation and necropsy for diagnosing fractures was evaluated by calculating the Kappa-value and performing McNemar’s Chi-square test (proc freq, SAS Institute). The results were interpreted as described by Dohoo et al. [51].

Association between deviation and fracture was evaluated doing a simple chi-square test (proc freq, SAS Institute). However, due to the fact, that data could be clustered on the herd level, a logistic regression with random intercept was used to account for the clustering effect. Flock-id and deviation*flock-id were included as random effects. Fracture was defined as the dependent variable, and deviation was the explanatory variable (proc glimmix, SAS Institute). To present the results, prevalence of fractures for each level of deviation was estimated, using the LS-means-option. The association between deviance and production system was analysed in an ordinal regression with a cumulative logit link, with deviance as the dependent variable and production system as the explanatory variable. Flock-id was included as a random effect.

The effect of production-system on callus formation were analysed in an ordinal, logistic regression-model (proc glimmix, SAS Institute). Only birds without new fractures were included in the analyses. The dependent variable was categorized on an ordinal scale, with no callus as the lowest category, minimal as the intermediate and moderate to severe callus as the highest category. The explanatory variables were production system and number of fractures. Flock was modelled as a random variable.

Body weight and production system: data were analysed in a generalized, linear mixed model using a logit transformation of the dependent variable and assuming a binomial distribution (proc glimmix, SAS Inst.). The dependent variable was fracture (yes/no) and explanatory variables were production system and weight. Possible interactions between the two were explored. Flock was introduced as a random effect, and the effect of weight could vary randomly between flocks. The effect of weight was modelled using an increase of 100 grams.

For, evaluation of the effect of production system and weight on the number of fractures, the data were analysed in a generalized, linear mixed model using a log transformation of the dependent variable and assuming a Poisson distribution. The dependent variable was number of fractures and explanatory variables were the production system and body weight. Possible interactions between the two were explored. Flock was introduced as a random effect, and the effect of body weight could vary randomly between flocks.

To evaluate the risk factor potential/effect of production parameters at given time points during the production cycle, a generalized mixed random model was applied under the assumption of Poisson distribution on log transformed data. In the model, the effect from production system and body weight was included. Estimated daily egg weight/day/hen is calculated as number of eggs per hen per day (egg yield) multiplied by the average egg weight for the given time point in the flock. All non-significant factors were excluded resulting in a model including estimated egg weight/day/hen at onset of lay, body weight at depopulation, age in weeks at depopulation and production type. The estimated daily egg weight was calculated as a function of egg yield and egg weight. For significant factors, least square means (LS-means) were estimated. LS-means estimates are to unbalanced designs what arithmetic means are to balanced designs. They represent the mean for the effect under investigation, for a subject, that is an average subject for all other factors in the model. This makes them especially suited for presenting data from complex models.

## Results

### Production data

Among the 40 flocks, production data was available from 24 flocks (Table 4), no production data from the three parent flocks was available.

**Table 4:** Overview on production data from 24 flocks.

	N	Min.	Max.	Mean	Std. Dev.	SEM	Median	25% Percentile	75% Percentile
**Age (weeks)**									
Onset of lay	24	20	24	21.29	0.955	0.195	21	21	22
Peak production	24	28	58	36.67	6.722	1.372	35	32	41
End of lay	24	60	88	77.21	6.058	1.236	77	76	81.75
**Egg yield (%)**									
Onset of lay	24	52.1	89	67.05	10.960	2.238	66.9	56.48	76.15
Peak production	24	92.4	99	96.87	1.695	0.346	97.35	96.13	98.08
Week 45	23	91	98.3	95.29	1.733	0.361	95.5	94.4	96.4
Week 50	24	89.7	98.2	94.55	2.154	0.440	95.25	93.7	95.9
Week 70	21	82.3	92.9	88.08	3.247	0.709	88.7	85	90.2
End of lay	24	23	93	72.36	20.790	4.244	80.45	67.93	86.28
**Egg weight (g)**									
Onset of lay	24	44	51.9	47.64	2.134	0.436	47.6	45.73	49.2
Peak production	24	58.6	64.6	61.92	1.658	0.339	62.25	60.95	63.05
Week 45	23	59.9	65.1	63.19	1.227	0.256	63.2	62.5	63.8
Week 50	23	60.7	65.2	63.57	1.101	0.230	63.6	63.2	64.5
Week 70	21	61.9	67.9	65.61	1.563	0.341	65.7	64.85	66.6
End of lay	24	57.2	69.1	65.70	2.565	0.524	66.15	64.4	67.35

### Fracture identification

Fracture identification was performed both by palpation of the intact carcass prior to necropsy and by necropsy of each bird (Table 5). A significant difference in the detection level was observed between necropsy and palpation (McNemar’s test, Chi square = 121.4703, DF = 1, *P* <0.0001) and the Kappa Coefficient of agreement was calculated to 0.7195 (95% CI: 0.6928–0.7462). Fractures with no or minimal callus were close to impossible to detect by palpation. Sensitivity of palpation in relation to necropsy was 0.9289 and specificity was 0.8840. Positive predictive value was 0.9796 and the negative predictive value was 0.6741.

**Table 5:** Contingency table KBF detected by palpation and necropsy.

		Necropsy		
		Fracture	No fracture	Total^a^
**Palpation**	Fracture	3799	79	3878
	No fracture	291	602	893
	Total	4090	681	4771

^a^ Males included.

None of the male birds had any signs of KBF and they were not included in further analysis.

Deviations >0.5 cm were present in 1702/4749 (35.8%) of the birds, of which 1583/4093 (38.7%) also had fractures while 119/656 (18.1%) had no fractures (Table 6). The relative risk of having a keel bone fracture when also having a moderate to severe keel bone deviation (>0.5 cm) was 1.129. The simple chi-square-statistic and the logistic regression both found an association between deviation and fracture to be significant (p<0.0001). The random effect of deviation between herds was non-significant (p = 0.33), indicating that the association between fracture and deviance is the same across herds.

**Table 6:** Association between deviation of keel bone and fracture across all flocks and between deviation and production system.

Deviation	Fracture^a^			Logistic regression	Production system				
	No fracture (n, (%))	Fracture (n, (%))	Total	Prevalence (CI.)^b^	Enriched cages (n, (%))	Parent stock (n, (%))	Barn house/Aviary (n, (%))	Organic/Free range (n, (%))	Total
x≤0.5 cm	537	2510	3047		749	320	1100	887	3056
	17.6%^c^	82.4%		89.9% (84.9% - 93.4%)	56.7%^d^	94.4%	61.6%	67.4%	
0.5<x≤1 cm	95	984	1079		382	15	415	268	1080
	8.8%	91.2%		93.7% (89.9%-96.1%)	28.9%	4.4%	23.3%	20.4%	
x>1cm	24	599	623		189	4	270	161	624
	3.9%	96.1%		96.9% (94.4%-98.3%)	14.4%	1.2%	15.1%	12.2%	
Total	656	4093	4749		1320	339	1785	1316	4760

^a^ Based individual birds.

^b^ Based on flock prevalence of KBF; CI: confidence interval.

^c^ Percentages within deviation categories.

^d^ Percentages within production system categories.

The apparent difference between the observed prevalence on the individual level and the results from the logistic regression is due to the fact that the results from the logistic regression is an average prevalence of fractures for birds with a certain deviance on the flock level. This is not necessarily the same as the effect on the individual level, because data are unbalanced. Results from the ordinal regression showed a significant difference between production forms (p = 0.0002) and a significant, random flock effect (p<0.0001). Parent flocks had a significantly lower deviation than the other three production systems (p = 0.0091).

Birds with severe deviations of the keel and breast muscles often had a saddle like appearance involving all sections of the keel bone resulting in extensive deformation of the ventral part of the bird.

### Production system

**Fig 1:**
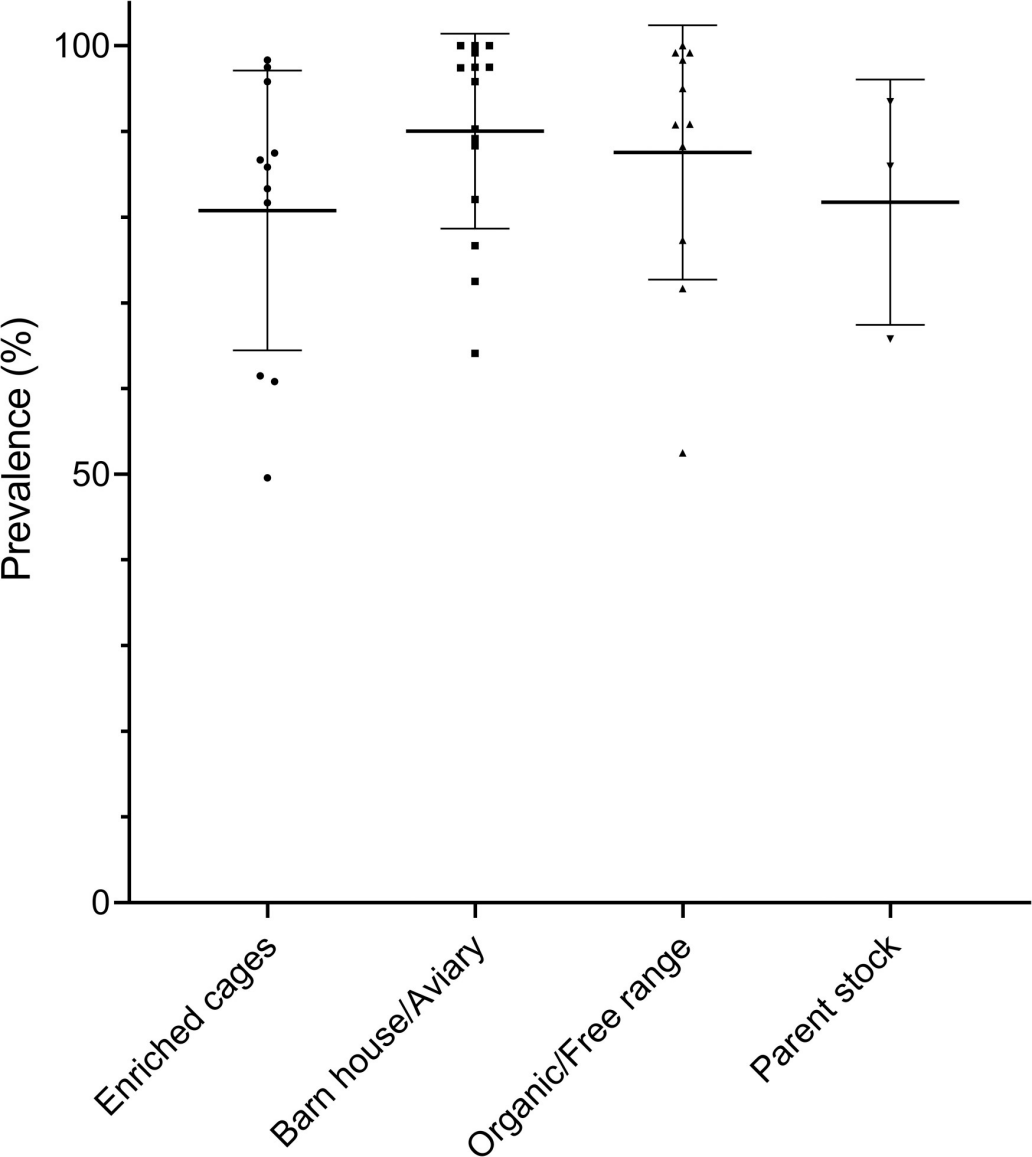
Scatter plot of the flock prevalence of KBF within each production system. Lines and error bars represent mean and standard deviation.

The overall (global) prevalence of KBF was 86.16% (Table 7), with global keel bone fracture prevalences ranging between 80.71–90.07% between production systems. Comparing the global prevalence between the four production systems, regardless that the birds originated from different flocks, a significant difference was observed (*P* <0.0001). There is however considerable variation in the individual flock prevalence (Fig 1). Taking the flock variation into account, by adding flocks into the model (Table 7), no differences were observed between the estimated mean prevalence of each production system (*P =* 0.19), however the flock effect was significant (*P* <0.0001).

**Table 7:** Global and estimated mean prevalence of KBF within in production systems.

Production system	No fracture		Fracture		Total^1^	Estimated Mean Prevalence^2^(%)	Lower Mean (%)	Upper Mean (%)
	Number of birds	Global prevalence (%)	Number of birds	Global prevalence (%)				
Enriched cages (n = 11)	255	19.29	1067	80.71	1322	86.32	72.35	93.84
Barn house/Aviary (n = 15)	177	9.93	1605	90.07	1782	95.29	90.07	97.83
Organic/Free range (n = 11)	164	12.46	1152	87.54	1316	93.83	85.87	97.44
Parent stock (n = 11)	62	18.45	274	81.55	336	84.88	51.26	96.77
Total	658	13.84	4098	86.16	4756			

^1^ No males were included in the analysis.

^2^The estimated mean prevalence obtained from a generalized mixed model after including a random flock effect.

The number of fractures in each bird differed between productions systems (*P* = 0.01) (Table 8). Hens from enriched cages and parent stock flock had the lowest number of fractures. More than 43% of hens from barn housed/Aviary or organic/free range flocks had 4 or more fractures at end of lay.

**Table 8:** Distribution of the number of fractures (%) in each hen within a production system (n = 4639 hens in 39 flocks).

Production system	Number of fractures per hen(%)					Mean No. fractures	Standard Error Mean	Lower Mean	Upper Mean
	0	1	2	3	≥4				
Enriched cages	19.29	22.24	22.16	16.34	19.97	1.8091	0.2128	1.4366	2.2782
Barn house/Aviary	8.11	10.69	17.3	14.47	49.43	2.4782	0.2902	1.9698	3.1177
Organic/Free range	12.46	13.22	15.12	15.96	43.24	2.7753	0.2875	2.2651	3.4003
Parent stock	18.45	33.33	18.16	16.96	13.10	1.5075	0.3406	0.9681	2.3475

### Fracture morphology, characteristics and presence of callus

**Fig 2:**
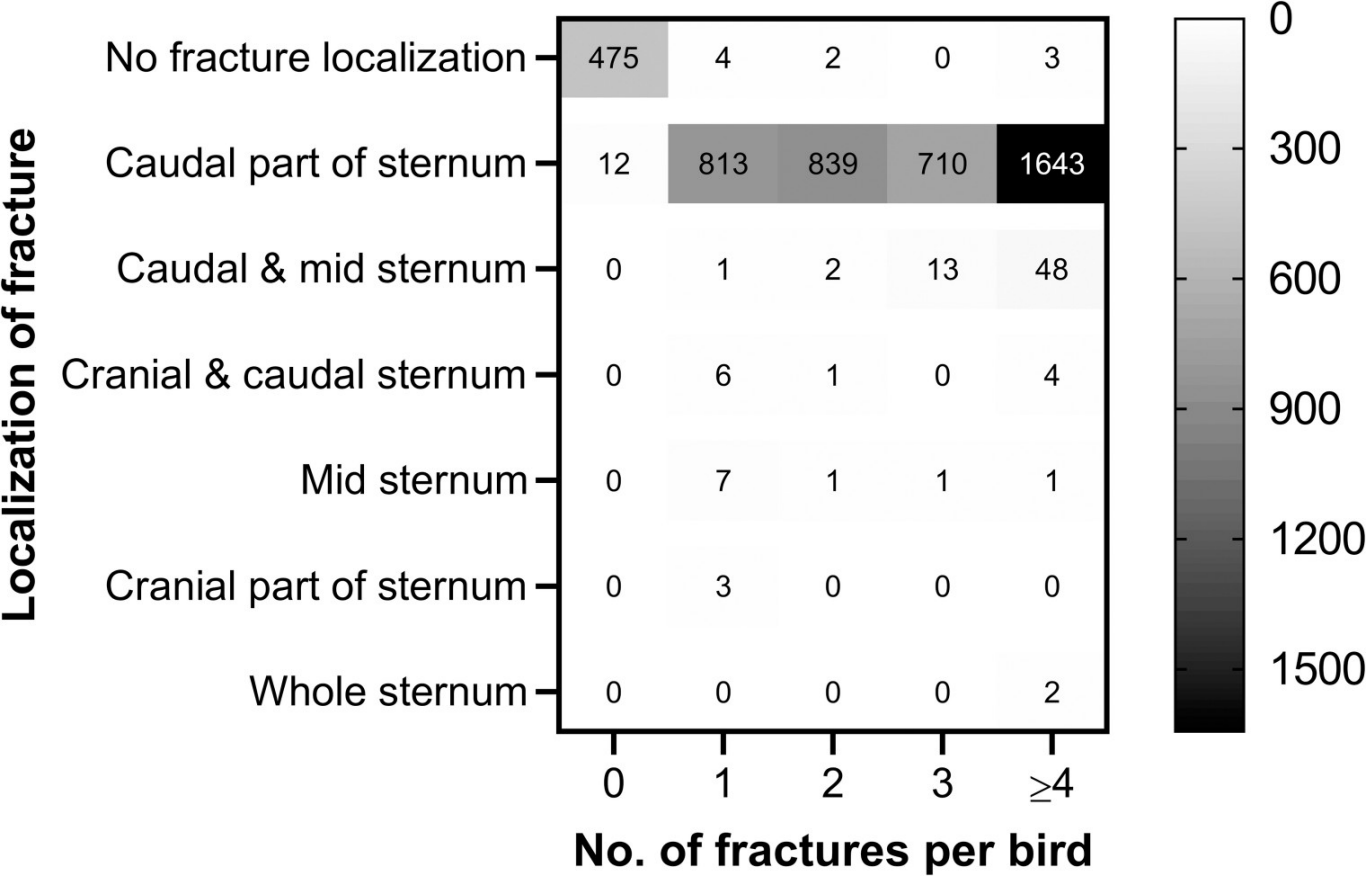
Heat map of the localization of KBF in relation to how many fractures detected in each hen (n = 4591). Values in the heat map cells represent the hen count.

In more than 96% of the hens with keel bone fracture, the fractures were solely present at the caudal tip of the keel bone (Table 9). The middle part of the sternum was the second most commonly involved part involving 1.85% (76/4104) of the hens. Due to the obvious differences in fracture localization regardless of the number of fractures per bird (Fig 2) no p-values were calculated.

**Table 9:** Distribution (%) of fracture localization in end of lay hens (n = 4591). The distribution is presented as percentages within columns.

Localization of fractures	No. of fractures per bird (%)				
	0	1	2	3	≥4
No fracture localization	97.54	0.48	0.24	0.00	0.18
Caudal part of sternum	2.46	97.48	99.29	98.07	96.59
Caudal & mid sternum	0.00	0.12	0.24	1.80	2.82
Cranial & caudal sternum	0.00	0.72	0.12	0.00	0.24
Mid sternum	0.00	0.84	0.12	0.14	0.06
Cranial part of sternum	0.00	0.36	0.00	0.00	0.00
Whole sternum	0.00	0.00	0.00	0.00	0.12

**Fig 3:**
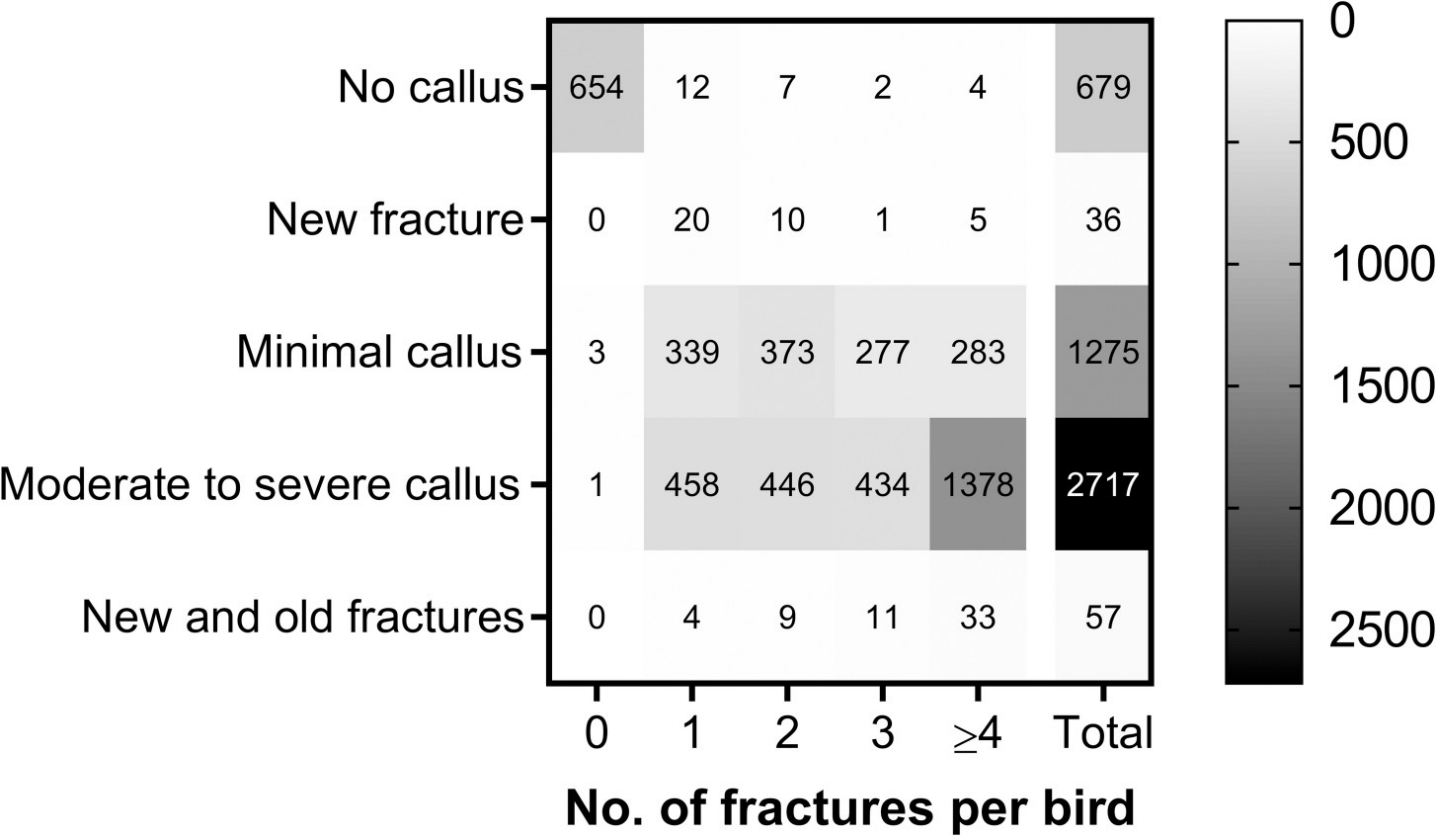
Heat map of the degree of callus formation in relation to the number of fractures detected in each hen (n = 4766). Values in the heat map cells represent the hen count.

Callus formation is a consequence of fracture repair. The degree of callus formation is dependent on the stability of the fracture, thus the more stable or immobilized a fracture is the less callus will develop outside the fracture line. In 99.4% of hens with no keel bone fracture no callus were observed (Table 10, Fig 3). A small number of the hens with fractures (36 hens, 0.76%) had fresh fractures, which were characterized by sharp fracture ends with non-union and varying degree of haemorrhage. The vast majority of these hens presented one or two fractures (Table 10, Fig 3). Of the hens with fractures, most of them presented various degrees of callus formations on the keel bone surfaces. About one quarter of the hens (26.8%) displayed minimal amounts of callus despite having at least one keel bone fracture. In the group of hens with minimal levels of callus, approximately 40% of the hens with 1–3 fractures had almost no callus around the fracture lines. In cases with ≥4 fractures with minimal callus, the dorsal (visceral) face of the keel bone with the consecutive rows of parallel fracture lines often looked like a washboard. More than half of the total number of hens investigated (57%) presented moderate to severe levels of callus in close relation to the fractures, with a trend that 80.9% of the hens with ≥4 fractures showing pronounced callus formation. These keel bones often showed a club-like appearance due to excessive amounts of callus at the caudal 3–4 cm of the keel bone.

**Table 10:** Association between callus characteristics and number of fractures per bird (n = 4758) or production system (n = 4760). The distribution is presented as percentages within columns.

Callus characteristics	No. of fractures per bird (%)					Production system (%)			
	0	1	2	3	≥4	Enriched cages	Barn house/Aviary	Organic/Free range	Parent stock
No callus	99.39	1.44	0.83	0.28	0.24	19.98	10.26	12.38	20.35
New fracture	0.00	2.40	1.18	0.14	0.29	0.30	1.18	0.61	0.88
Minimal callus	0.46	40.63	44.19	38.12	16.63	58.52	14.58	12.76	21.24
Moderate to severe callus	0.15	55.05	52.73	59.94	80.90	20.36	72.91	72.59	56.05
New and old fractures	0.00	0.48	1.07	1.52	1.94	0.83	1.07	1.67	1.47

Table 10 shows the level of callus formation dependent on production system and number of fractures. The results from the ordinal, logistic regression showed a significant effect of the number of fractures (p<0.0001) and the production-system (p = 0.0058). The significant effect of production from could be explained by the low callus-formation in birds from enriched cages (p = 0.0016). There were no significant differences between the other 3 production-systems.

The significant effect of number of fractures were to a large extent explained by birds without a registered fracture (Table 10). Data were re-analysed by excluding birds without a fracture. The effect of production-system was still significant, and there was an increase in callus-formation with an increasing number of fractures (p<0.0001), so the effect was also present in birds with a registered fracture. More fractures resulted in more callus formation.

### Body weight and age at depopulation

The effect of body weight (BW) at the day of depopulation was investigated. Assuming a random flock effect results in a declining KBF prevalence with higher BW (Table 11).

**Table 11:** The prevalence of keel bone fracture in end of lay hens according to BW at depopulation (250 g intervals) (n = 4756).

Body weight (g)	Prevalence (%)	Lower Mean (%)	Upper Mean (%)
≤1375	94.04	89.29	96.76
1376–1625	93.75	89.79	96.24
1626–1875	91.67	86.68	94.90
1876–2125	91.23	85.43	94.86
>2125	89.80	80.25	95.02

The interaction between weight and production system was overall significant (p = 0.05), indicating that the effect of weight is different between production systems. There was a significant random variation of the effect of weight between flocks.

**Fig 4:**
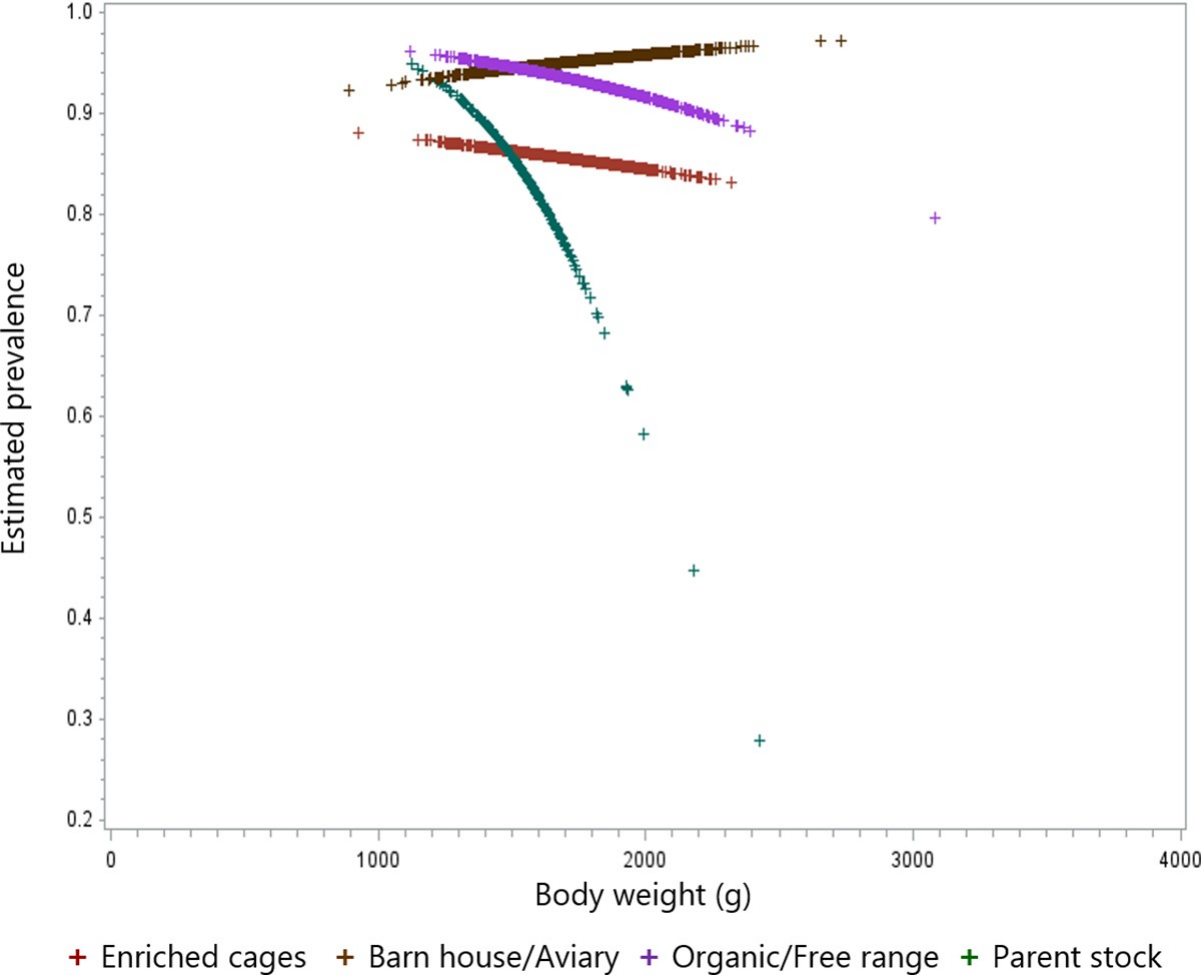
Scatter plot of the estimated prevalence of KBF for each hen as a function of the body weight at depopulation.

The interpretation odds ratios in models with an interaction are difficult (Table 12). Fig 4 shows the prevalence of fractures for each production system, dependent on weight.

**Table 12:** Overview of the effects of body weight (BW) and the interaction of BW and production system on the probability of having KBF. Results from the generalized, linear mixed model.

Variable	Estimate	P- value	Lower	Upper	Odds Ratio (OR)	Upper	Lower
Intercept	4.2929	0.0001	2.1627	6.4231			
100 gram increase BW (100 g)	-0.09498	0.1361	-0.2200	0.03006	0.91	1.15	0.80
Enriched cages	-2.0179	0.1358	-4.6775	0.6417	0.13	1.15	0.01
Parent stock	1.9563	0.3759	-2.3895	6.3021	7.07	1.46	0.09
Barn house/Aviary	-2.3464	0.1074	-5.2068	0.5139	0.10	1.11	0.01
Organic/Free range	0	.	.	.	1		
Interaction							
100 g* Enriched cages	0.06606	0.4170	-0.09397	0.2261	1.07	1.52	0.91
100 g* Parent stock	-0.2018	0.1553	-0.4803	0.07680	0.82	1.17	0.61
100 g* Barn house/Aviary	0.1548	0.0787	-0.01783	0.3274	1.17	1.08	0.98
100 g* Organic/Free range	0	.	.	.	0.91	1.15	0.80

It showed, that the effect of weight is pronounced for parent stock. Individual analyses per production system showed, that the effect of weight was only significant for parent stock (p = 0.02) (Fig 4, Table 12). This means that heavy birds have significantly lower prevalence of KBF in this production system. This effect of body weight cannot be excluded in organic/free range or caged flocks. The random effect of weight across flocks was significant (p = 0.03), indicating that the effect of weight differs between flocks, even when the production system was taken into account. Thus, there was a reduced prevalence of KBF in heavier birds.

The number of fractures and body weight at depopulation was also significantly correlated, meaning that heavier birds had fewer fractures than lighter birds. The interaction between production system and weight was insignificant (p = 0.16). Table 13 shows the parameter-estimates, p-values and relative risks.

**Table 13:** Overview of the effects of body weight (BW) and the interaction of BW and production system on the number of KBF. Results from the generalized, linear mixed model.

Effect	Estimate	P- value	Lower	Upper	Relative risk (RR)	Lower	Upper
Intercept	1.5189	< .0001	1.2382	1.7997			
Weight in 100 grams	-0.03631	< .0001	-0.05091	-0.02172	0.96	0.95	0.97
Enriched cages	-0.3048	0.0391	-0.5943	-0.01529	0.73	0.55	0.98
Parent stock	-0.3337	0.1389	-0.7757	0.1083	0.71	0.46	1.11
Barn house/Aviary	0.04506	0.7470	-0.2287	0.3188	1.05	0.79	1.37
Organic/Free range	0	.	.	.	1	.	.

The overall effect of production system was significant (p = 0.03). Enriched cages and parent stock flocks had lower relative risks than Organic/Free range and Barn house/Aviary flocks.

**Fig 5:**
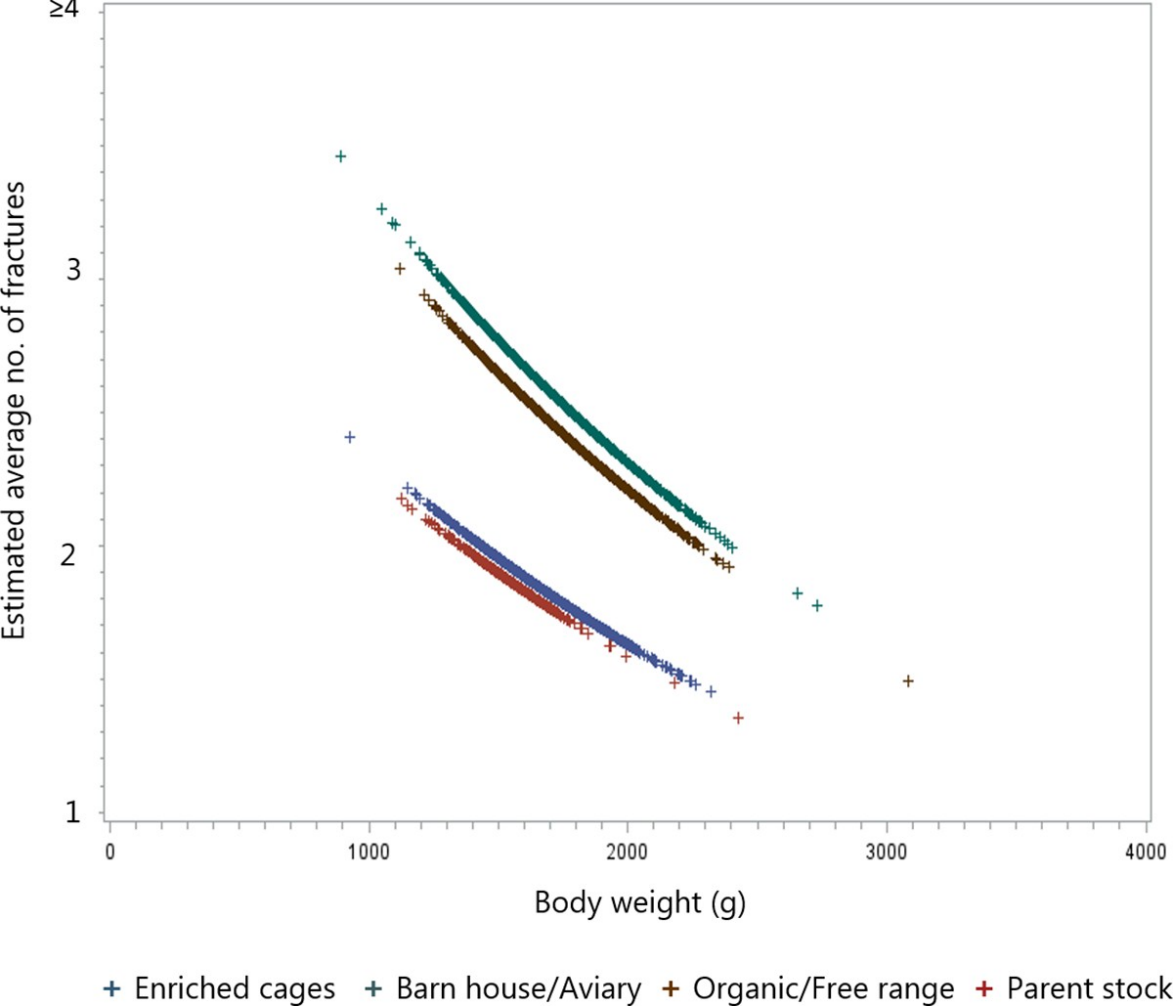
Scatter plot of the estimated average number of KBF for each hen as a function of the body weight at depopulation.

For every 100-gram increase in weight, number of fractures will drop by 4%. In the model, this effect could vary between flocks. This variation was significant (p = 0.004), indicating that the effect is not the same in all flocks. Fig 5 shows the effect of production system and weight.

**Fig 6:**
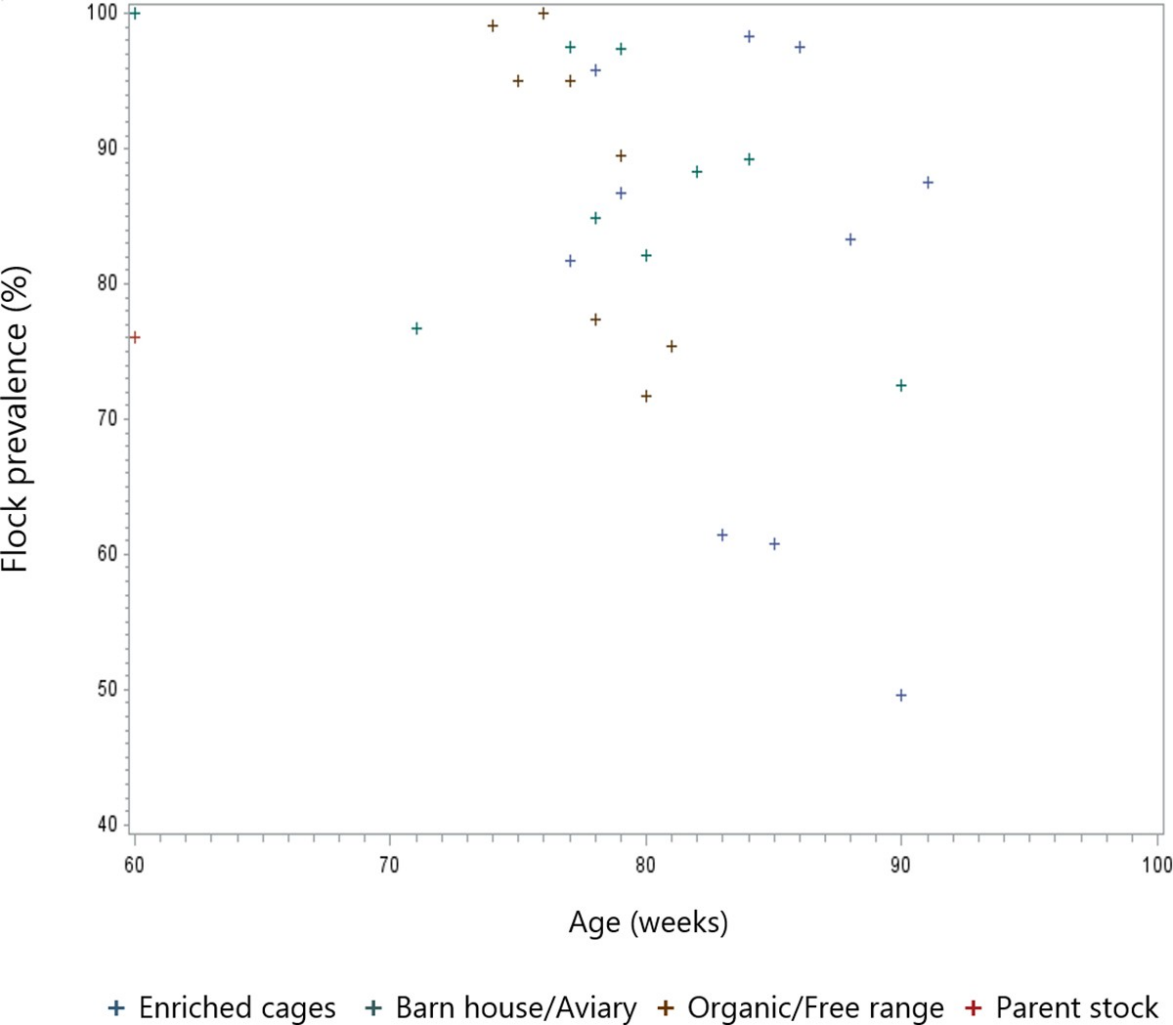
Scatter plot of the flock prevalence in relation to the flock age at depopulation.

When looking at the flock prevalence in relation to age at depopulation (Fig 6) no significant effect of age could be observed, however a trend towards a lower prevalence with higher age at depopulation was observed.

### Risk factor identification

The risk factor analysis of the production data, based on a generalized mixed random model with the assumption of Poisson distribution on log transformed data, revealed that age at onset of lay, estimated daily egg weight at onset of lay, body weight at depopulation and production system influenced the prevalence of KBF (Table 14). This means that for every week of age that the onset of lay is delayed the risk of developing KBF at the end of the production cycle is reduced by 12%. The estimated daily egg weight at onset of lay also has an effect on the occurrence of KBF. For every gram the egg weight at onset of lay is increased the number of fractures increased by 3%. An increase in body weight of 100 g at depopulation reduced the prevalence of KBF in end of lay hens by 3%. When including body weight, age at onset of lay, estimated daily egg weight at onset of lay, hens from barn housed/aviary flocks have the highest prevalence and caged hens had the lowest.

**Table 14:** Incidence ratios for risk factors with significant effect on the presence of keel bone factors in end of lay hens (n = 24 flocks).

Factors	Incidence ratio	Lower Confidence level	Upper confidcence level	*P*-value
Age at onset of lay (weeks)	0.88	0.78	0.98	0.0261
Estimated daily egg weight/hen at onset of lay (g)	1.03	1.01	1.05	0.0036
Body weight at depopulation (100 g increase)	0.97	0.96	0.99	0.002
Enriched cages	0.59	0.45	0.79	0.0004
Barn house/Aviary	1.21	0.91	1.59	0.1858
Organic/Free range	1			.

A similar pattern was observed when estimating the LS means of the data for the factors above (age at onset of lay, estimated daily egg weight at onset of lay, body weight at depopulation and production system) (Table 15). If onset of lay was at 22–24 weeks of age, the lowest estimated average number of KBF was approximately 1.9 fractures/hen by the end-of-lay. Similarly, if the estimated daily egg weight per hen was 25 and 30 at onset of lay, this was predicted to result in less than 2 fractures/hen by the end-of-lay. Birds with a body weight of 2500 g have the lowest average number of fractures, whereas light to midweight hens had the highest number of fractures. Additionally, the number of fractures per hen in the non-caged production systems was almost twice the estimated number in caged birds.

**Table 15:** Estimated number of KBF per hen (least squares means) for different levels of potential risk factors.

	Least Squares Means			
	Estimated average No. fractures/hen	Standard Error Mean	Lower Mean	Upper Mean
**Production system**				
Enriched cages	1.4543	0.1689	1.1580	1.8263
Barn house/Aviary	2.9300	0.2879	2.4165	3.5527
Organic/Free range	2.5943	0.3319	2.0187	3.3341
**Age at onset of lay (weeks)**				
20	2.4503	0.2768	1.9635	3.0577
21	2.7789	0.2644	2.3059	3.3488
22	1.8904	0.1942	1.5455	2.3122
24	1.9131	0.4700	1.1818	3.0971
**Body weight at depopulation (g), 500 g intervals**				
1000	2.3036	0.3928	1.6489	3.2181
1500	2.3408	0.1691	2.0316	2.6969
2000	2.2243	0.1664	1.9208	2.5757
2500	2.0532	0.3276	1.5016	2.8073
**Estimated daily egg weight/hen at onset of lay (g)**				
25	1.6685	0.1818	1.3476	2.0659
30	1.9458	0.2178	1.5623	2.4234
35	3.1913	0.3387	2.5917	3.9297
40	2.8499	0.3243	2.2800	3.5622
45	2.4993	0.5005	1.6876	3.7014

Apart from the results described above, no other significant findings from the production data could be identified. As mentioned above, the distribution of the different hen lines was skewed in favour of Lohmann LSL Classic hens. However, analysis including solely hens from Lohmann LSL Classic flock did not reveal any other patterns than already described above.

## Discussion

The data presented in this paper are, to the best of our knowledge, the first analysis of the occurrence of KBF in hen production, it relied primarily on post mortem findings combined with retrospective production data from the investigated flocks. The findings in this study span from evaluation of diagnostic methods used for detection and identification of the KBF to the analysis of production results from different time point in the production cycle and the potential association with the presence of KBF in hens at the end of lay. Unfortunately, it was not possible to obtain production data from all the flocks included in the study. This may be explained by the fact that it is voluntary to join the common database for the egg producers in Denmark. In addition, it was not possible to obtain production results from the three participating parent flocks due to breeder bird company policy. The observed skewness in hen lines was unexpected, thus rendering further analysis on the significance of the hybrids on the prevalence of KBF meaningless. Within the last couple of years, the organic Danish egg producers have switched from brown to white layers due to a higher egg yield per housed hen. This change in combination with a 3-year period of data collection may explain the overrepresentation of Lohmann LSL Classic hens in the present study.

Palpation has until now been the most commonly used diagnostic method to identify KBF in layer hens in the field. There is however increasing evidence that diagnosis by palpation underestimates the true prevalence in a flock [10,24,29,32]. Although fracture identification by palpation is very often in agreement with the necropsy identified fracture presence (high Kappa value), the difference in the levels of fracture identification is highly significant, thus meaning that using palpation alone is underestimating the true prevalence. The high Kappa value should also be interpreted with caution as agreement analysis is most reliable for prevalences in the range 20–80% [51], and we observed an overall KBF prevalence of 86.2%. The underdiagnosis of fractures with no or minimal callus by palpation is, in our experience, a very plausible explanation for the low prevalence reported from caged birds in earlier studies. These non-productive fracture lines (i.e., not accumulating callus for stabilisation of fracture) are very difficult to identify by palpation. Just like the vast majority of all KBF, they are located on the dorsal (visceral) aspect of the caudal tip of the keel bone. In order to palpate them, the operator has to put a finger deep behind the tip of the keel. We experienced that these fractures were very difficult to identify, even when the hens were dead, and therefore no muscular tone was present and we had unlimited time to perform the palpation. The time needed for recognizing a fracture with no callus is not well tolerated by live hens as it may pose discomfort for the hen if restrained and palpated deeply behind the rib curvature. The lack of identification of fractures with minimal callus by palpation has also been described by other authors [4,10,27,30], where radiography or dissection highlighted the discrepancy between diagnostic methods. From a pathological perspective, we found it striking that the amount of callus seemed much less in birds from caged flocks compared to the non-caged flocks. This was also reflected in the callus distribution profiles, where caged hens had significantly less callus compared to the cage-free systems. The fact that about half of the caged hens with KBF have minimal callus deposition will most likely lead to a considerable underdiagnosis of KBF in caged birds if palpation is used as evaluation method. The evident discrepancy between evaluation methods is also highlighted in a recent systematic review by Rufener and Makagon [45], where they emphasized the necessity of reliable and standardized methodology for evaluation of keel bone fractures.

Deviations have previously been associated with fractures [17]. In the present study there was an increased risk of having moderate to severe deviations (>0.5 cm) when also having fractures. The present study revealed that despite a considerable flock variation in the prevalence of deviations a difference between production systems was observed, with parent stock having fewer birds with deviated keels. Regardless of the association between fractures and deviations, it is evident that the fractures, most often have a different localization on the keel bone than the deviations, with the caudal third being most often involved [4,10,11,19,24]. With the perspective of localization alone it may be likely that deviations and fractures have different causes and thereby also different pathogenesis. It is possible that the high prevalence of fractures may blur the differentiation of these.

In the present study, the overall prevalence across the 40 flocks was among the highest reported regardless of production system [3–11]. Previously, higher prevalence was found in non-caged flocks, we could however not see the same trend as we found no difference between the production systems. What we observed was a considerable flock variation regardless of production system. When looking at the number of fractures per bird, we observed differences betweeen the production systems. It is striking that almost half of the birds from barn housed/Aviary or organic/free range flocks had at least 4 fractures at the end of the laying cycle. The reason for this difference between the systems is not yet known, as the number of fractures per bird has not received much attention before, but it is known that birds often have multiple fractures [10,29,52]. It is likely to assume that the chance of palpating fractures are higher when the birds have more fractures. Consequently, this could contribute to the prevoiusly observed variation in fracture occurrence between production systems resulting in higher rates in non-caged systems where palpation was the diagnostic method. Other research has previously evaluated “Keel bone severity” in both caged and non-caged systems [4,29,40,41]. This severity was based on radiographic evaluation or dissection and criteria for assessing the severity was the total amount of keel bone affected by fracture, but details on the fracture complexity (e.g. number of fractures, gaps in fracture lines, amount of callus, sclerosis, etc.) was not included *per se* but contributed as a subjective assessment to the total aggregate fracture severity.

We can also see that about half of the birds from parent stock flock have ≤1 fracture per hen. It should be noted that these three flocks were all culled at 60 weeks of age, the youngest among the 40 flocks. A possible explanation for this could be that the expected performance goals for parent birds with regards to egg laying are approximately 5% lower for parent stock compared to layer flocks at 72 weeks of age [53,54]. Other factors that may play a role could be the different management of parents (e.g. stocking density, housing equipment, presense of males). It is known that the prevalence is seen to increase with age, but it seems that the prevalence levels out from around 50 weeks of age [45], with some differences between white and brown hen lines [40,41].

Although small in numbers, we were able, for the first time, to report observations on KBF in male birds from commercial layer lines. In the examined males, all coming from parent stock flocks, no fractures was identified. These findings are in line with the observations on Red Jungle Fowl roosters [55]. Whether this has to do with weight or the fact that they do not lay eggs is highly relevant to address.

It has been reported that the majority of the KBF are simple transverse or oblique fractures [10]. This is also supported by histopathological characterization of KBF by Thøfner et al. [19], which included a subset of keels sampled from the hens included in the present study. Although no systematic recording of fracture line pattern for each fracture was performed in the present study, we are of the impression that the fracture pattern is in agreement with previous observations [10,19].

The caudal tip (1/3) of the keel bone seems to be a predilection site for the KBF [4,10,11,19,24]. In the present study, the localization of the keel bone fracture strongly supports this notion. The observed level (>99%) is however somewhat higher than recently reported (79%) by Baur and co-workers [10] but may be due to differences in the type of production systems across countries [19].

In the present study, the amount of callus formation observed indicates a relationship between considerable callus formation around the fractures and the number of fractures per bird as well as the production system. Recently it was demonstrated that radiographic assessment [29] is a reliable tool for assessing the severity, including both number of fractures and amount of callus. This is in line with our observations, showing multiple fractures (≥4) often are accompanied by excessive amounts of callus and that birds with few fractures often have minimal callus.

The relationship between body weight and the occurrence of KBF has not received much attention in previous studies. A Swiss study on three different layer lines at the time of end-lay observed that heavy hens had the highest fracture severity [35]. This finding is in clear contrast to our findings, where we have observed a significant decrease in number of fractures per bird with increased body weight regardless of production type. Furthermore, we also observed a significant decrease in fracture prevalence with increased body weight in hens from organic/free range and parent stock flocks. Wei et al. [56] showed that 27 and 42 weeks old birds with fractures were significantly lighter than birds with no fractures, thus supporting our findings.

Although not significant, the age at depopulation in the present study may indicate that flocks with the highest age have a lower KBF prevalence at depopulation. Whether this is due to more robust hens and thus an increased longevity needs further investigation.

Risk factor identification for the development of KBF are limited. Part of the explanation for this may be as summed up by Jung et al. [20], where it was proposed that keel bone injuries, both deviation/damage and fractures have a multifactorial origin. Although observations on both keel bone deviations and fractures have been collected in this cross-European study, the palpation assessment of the fractures at the distal part (1.5 cm) of the keel was excluded from data analysis due to poor inter-rater agreement, and therefore the risk factor analysis in that study may be directed towards keel bone deviations rather than fractures. In the present study, the vast majority of fractures were localised at the distal tip, as discussed above, and the fractures in caged birds had often next to no callus, thus were impossible to palpate in live birds. This observation underlines the disagreement between palpation and necropsy in the assessment of fracture identification. The lack of true risk factor identification for keel bone fracture development, to some extent at least, may be due to lack of separation of deviations, damage and fractures, which are likely to have different pathogeneses. Just like it has been documented that perches have a significant role in the development of deviations at the rim of the keel [14,21,22]. As no single cause of keel bone fracture development has yet been identified, the efforts in risk factor identification have to some extent been inconclusive and argue for more research into understanding these fractures.

However, it is observed that high laying performance is associated with high prevalence of both keel bone damage and fractures [20,37], and thus is proposed as a risk factor. In the present study it was not possible to link the overall performance to higher fracture prevalence. A possible explanation for this may be that we were not able to obtain production data from all flocks included in the study. However, the effect cannot be excluded and may have been masked by the very high prevalence of KBF and maybe also by the relatively uniform and high performance levels at peak to 70 weeks. Further investigation into the details of the egg yield is needed [43]. There are several reports where a lower egg yield in individual hens has been linked to fracture development [40,57–59]. A recent study investigated the prevalence in the indigenous chicken, Red Jungle Fowl, and found that 1 out of 12 hens had KBF and none of 17 roosters investigated had fractures [55]. These indigenous chickens only produce 4–6 eggs per breeding season as opposed to modern layer which produces >300 eggs per production cycle [60].

The multifactorial origin has also received attention by Toscano et al. [43], where four areas, which need further investigation for unravelling of the causes of KBF, in particular, were identified. The areas are: the age at first egg, late ossification of the keel, predisposing bone diseases, and inactivity leading to poor bone health.

In the present study, we have demonstrated that heavier birds are less likely to develop fractures and they also have fewer fractures than lighter birds. Risk factor analysis revealed that this effect may be quantified meaning that the risk of developing KBF throughout the production period is lowered by 3% per 100 g increase in bodyweight. This is in contrast to the breeding goals over the last couple of decades where the hens have been bred to a smaller body mass in order to reduce the energy needed for maintenance and for a higher egg yield [61,62] with bigger eggs earlier in the laying period [62–64]. Thus, the modern hen is smaller in body size and lays more and bigger eggs and has a potential of an earlier onset of lay. We have demonstrated that a delay in onset of lay resulted in a lower flock prevalence of KBF at the end of the production. This is in agreement with earlier observations proposing early onset of lay as a risk factor for the development of KBF [36]. The magnitude of the relative risk of delay in onset of lay, based on the production data, was surprisingly large (12% per week). Further investigation seeking to confirm this effect is highly needed, also across different hen lines.

In the present study, we observed that the estimated daily egg weight (gram) at the onset of lay may be used as a predictor of the risk of high flock prevelance late in the production. The lower estimated daily egg weight at onset of lay results in fewer fractures in the flock. The risk increases by 3% per 1 gram increase in the estimated daily egg weight at the onset of lay. Thus indicating that big eggs in the early laying period may have a negative effect on the keel bones. The majority of the fractures develop during weeks 25–50 [7,17,45] with a peak at 35 weeks [10]. In the modern layer hen, this coincides with peak egg production.

Our findings suggest that the period around onset of lay is of particular interest in the development of KBF, both in regards to production parameters and the anatomy and physiology of the modern laying hen.

The similar level of prevalence in all production systems suggests a common cause of KBF involving the bird. As mentioned previously the birds have become smaller over the years and at the same time a higher egg production has been achieved. In addition, late ossification of the distal part of the keel bone has been suggested to represent a weak spot in relation to the development of KBF as most fractures are observed here [19]. It also has been demonstrated that the majority of fractures appear as if they develop from the inside of the bird [19]. The findings in this study concerning weight of the birds, egg weight and onset of lay support the hypothesis that pressure from the inside of the body cavity in small birds with a not yet fully ossified keel bone and producing large eggs biomechanically may affect the late ossified tip of the keel in a way that may result in fracture development.

## Conclusions

In conclusion, the study has, besides determining the prevalence of KBF in the Danish layer production, identified hen size, age at onset of lay and estimated daily egg weight at onset of lay to be major risk factors for development of KBF in the modern laying hen. Further research regarding this is warranted to strengthen the longevity and secure the welfare of laying hens.

## Supporting information

S1 File Flock prevalences and production data. The file contains data on flock prevalences on KBF and Prevalence of Number of fractures in each bird within production system as well as flock level production data.
(XLSX)

S2 File Raw data used for the statistical analysis. The file contains variable definitions and data with all registrations on bird level (one row represents one bird).
(XLS)
